# COMmunication with Families regarding ORgan and Tissue donation after death in intensive care (COMFORT): protocol for an intervention study

**DOI:** 10.1186/s12913-016-1964-7

**Published:** 2017-01-17

**Authors:** Julie E. Potter, Robert G. Herkes, Lin Perry, Rosalind M. Elliott, Anders Aneman, Jorge L. Brieva, Elena Cavazzoni, Andrew T. H. Cheng, Michael J. O’Leary, Ian M. Seppelt, Val Gebski, the COMFORT study investigators

**Affiliations:** 1 NSW Organ and Tissue Donation Service, South Eastern Sydney Local Health District, PO Box 486, Kogarah, 1485 NSW Australia; 2 Intensive Care Service, Royal Prince Alfred Hospital, Camperdown, NSW Australia; 3 Prince of Wales Hospital, East Wing Edmund Blackett Building, Randwick, NSW Australia; 4 Department of Intensive Care, Royal North Shore Hospital, St Leonards, NSW Australia; 5 Department of Intensive Care, Liverpool Hospital, Liverpool, NSW Australia; 6 Division of Anaesthesia, Intensive Care and Pain Management, John Hunter Hospital, New Lambton Heights, NSW Australia; 7 Department of Paediatric Intensive Care, The Children’s Hospital at Westmead, Westmead, NSW Australia; 8 Department of Intensive Care, Saint George Hospital, Kogarah, NSW Australia; 9 Department of Intensive Care, Nepean Hospital, Nepean, NSW Australia; 10 National Health and Medical Research Council (NHMRC) Clinical Trials Centre, Camperdown, NSW Australia

**Keywords:** Communication, Decision making, Family decision, Intensive care unit, Organ donation, Requester, Third party consent, Tissue and organ procurement

## Abstract

**Background:**

Discussing deceased organ donation can be difficult not only for families but for health professionals who initiate and manage the conversations. It is well recognised that the methods of communication and communication skills of health professionals are key influences on decisions made by families regarding organ donation.

**Methods:**

This multicentre study is being performed in nine intensive care units with follow-up conducted by the Organ and Tissue Donation Service in New South Wales (NSW) Australia. The control condition is pre-intervention usual practice for at least six months before each site implements the intervention. The COMFORT intervention consists of six elements: family conversations regarding offers for organ donation to be led by a “designated requester”; family offers for donation are deferred to the designated requester; the offer of donation is separated from the end-of-life discussion that death is inevitable; it takes place within a structured family donation conversation using a “balanced” approach. Designated requesters may be intensivists, critical care nurses or social workers prepared by attending the three-day national “Family Donation Conversation” workshops, and the half-day NSW Simulation Program. The design is pre-post intervention to compare rates of family consent for organ donation six months before and under the intervention. Each ICU crosses from using the control to intervention condition after the site initiation visit. The primary endpoint is the consent rate for deceased organ donation calculated from 140 eligible next of kin families. Secondary endpoints are health professionals’ adherence rates to core elements of the intervention; identification of predictors of family donation decision; and the proportion of families who regret their final donation decision at 90 days.

**Discussion:**

The pragmatic design of this study may identify ‘what works’ in usual clinical settings when requesting organ donation in critical care areas, both in terms of changes in practice healthcare professionals are willing and able to adopt, and the effect this may have on desired outcomes. The findings of this study will be indicative of the potential benefits of the intervention and be relevant and transferrable to clinical settings in other states and countries.

**Trial registration:**

Australian New Zealand Clinical Trials Registry (ANZCTR): ACTRN12613000815763 (24 July 2013). ClinicalTrials.gov: NCT01922310 (14 August 2013) (retrospectively registered).

**Electronic supplementary material:**

The online version of this article (doi:10.1186/s12913-016-1964-7) contains supplementary material, which is available to authorized users.

## Background

Organ and tissue transplantation is the definitive treatment for people with a wide range of end-stage organ failures. Escalating worldwide demand continues to drive efforts in many countries to increase the rate of deceased organ donation [1]. In Australia in 2011 the annual rate of deceased organ donation (15.1 donors per million population, dpmp) was below similar countries in the developed world such as the United Kingdom (UK) (17 dpmp), and international leaders such as the United States of America (USA, 25.9 dpmp), and Spain (35.9 dpmp) [2].

In New South Wales (NSW), Australia, consent for organ donation must be provided either from the patient while living or, when incapacitated, from the senior available next-of-kin (SaNOK): that is, a relative identified in line with an agreed family hierarchy [3]. In NSW the annual consent rate increased from 51% in 2011 [4] to 62% in 2013 [*unpublished 2014 NSW Organ and Tissue Donation Service (OTDS) data*], contributing to an annual national increase from 57% [5] to 61% [6] respectively. Other countries with similar health systems such as the UK, also reported a national consent rate of around 60% for 2013/14 [7]. Yet this consent rate is comparatively low in contrast to overwhelming positive public opinion in surveys of the UK and Australian populations with 90% and 69% respectively supporting donation and willing to become organ donors, describing predominantly altruistic beliefs on the topic [7–9]. Furthermore the consent rate decreased to 29% *[unpublished 2011 NSW OTDS data]* when families have been offered organ donation at the hospital and the patient had not previously registered their donation decision.

Physicians need effective communication skills when approaching families regarding end-of-life decisions, and skill enhancement has been advocated to maximise the consent rate for deceased donation [10, 11]. The difficulty of raising the subject of organ donation has long been recognised. North American research revealed Intensive Care Unit (ICU) physicians were poorly prepared to understand grief reactions, missed opportunities to provide emotional support, and failed to listen to families and support informed decision-making [12–14]. In Australia, a one-day donor awareness program designed to increase health professional’s understanding of organ donation and to provide skills to sensitively conduct family donation conversations has been available since 1994 [15], with intensivists reporting this training as adequate preparation [16]. Despite that training, ICU nurses, intensivists and specialist donation nurses may avoid raising the topic due to their own perceptions of a family’s grief, fear or guilt, or of adding to a family’s distress [17, 18]. However, a longitudinal study of 49 relatives in the UK reported that discussing organ donation did not increase families’ distress [19].

The approach and skill of the health professional making the donation request has been shown to be a key influence on families’ donation decisions [20]. In countries that lead in this field such as Spain and the USA, health professionals working as transplant coordinators or organ procurement coordinators receive specialised communication training and make the initial approach to families [10, 21, 22]. In Australia, the managing intensivist traditionally leads donation discussions with introduction to the donation specialist nurse (DSN) subsequent to verbal agreement to organ donation. DSNs are trained in organ donation activities, but not necessarily in leading the conversation [23]. Many intensivists only participate in organ donation discussions a few times per year, providing limited opportunities to practice the necessary specialised communication skills [16].

### ‘Best practice’ family approach

Intensive care medicine professional organisations and health authorities in Australia and the UK provide practice guidelines for communication between families and health professionals during end-of-life care, including organ donation decisions [23, 24]. In Spain and the UK a ‘family approach’ has been recommended with the requesting conversation planned between the managing team and a Specialist Nurse-Organ Donation [10, 25]. Ensuring families understand that the patient has died or that death is inevitable before donation is raised is a key feature. The initial approach to the topic of donation has been identified as a pivotal point in the process because families often make their donation decision at that time [26].

Effective communication within a structured multidisciplinary family meeting, also termed ‘family conference’, has been recommended for ICU physicians to facilitate informed decision making based on the anticipated wishes of the patient rather than those of the patient’s relatives [27–31]. Meetings ideally require multidisciplinary team planning, a private location and effective communication techniques such as the use of everyday language, listening and acknowledging relatives’ emotions or opinions and demonstrating compassion verbally and through non-verbal techniques [23, 32]. In the USA communication approaches have moved from a “neutral” position towards organ donation towards one of “dual advocacy”. This entails use of positive language, equally presenting the needs of the donor family and people on transplant waiting lists, assuming that most people would want to help others by donation [33, 34]. Training organ procurement coordinators (*n =* 22) in effective communication techniques for requesting organ donation increased the consent rate in participating hospitals by 9.2%, over 2 years [22, 35]. Families who were certain of their organ donation decision reported that health professionals providing them with clear information and emotional support were key factors in helping them make decisions with which they remained comfortable over time [36].

Studies conducted in North America have shown some donation decisions were later regretted, and that this occurred more frequently when the decision was to decline donation. For example, of 285 relatives interviewed an average of 13 ± 9 days after death, only 4% (6/147) who had agreed to donation would later have preferred they had declined. By contrast, of those who declined donation, 27% (37/138) later wished they had agreed [37]. Decisional regret, where relatives either regretted their decision or were unsure they would make the same decision again, persisted up to 10 months after bereavement. A study of 199 relatives interviewed eight to ten months after death revealed decisional regret was more evident in those who declined (42%; 19/45) than agreed to donation (9%; 15/154) [36]. Decisional regret was more likely when organ donation was raised before relatives were informed of the patient’s death, and when the first approach was by a health professional who managed the patient’s care, before a formal request from a separate organ procurement team [37].

### Specialised communication training

In Australia, training in specialised communication for health professionals who offer donation has been a key component of a national reform agenda [38]. A national program of specialised training in family-centred communication regarding organ donation, developed in collaboration with the Gift of Life Institute (Philadelphia, USA), was introduced in October 2011 [39]. The revised program delivered in two modules over three days, incorporated face-to-face presentations of theory followed by practical training with role-play exercises [40]. This approach — the Organ and Tissue Authority (OTA) Family Donation Conversation (FDC) core and practical modules – (see Additional file 1) has been adopted as ‘best practice’; intensive care specialists and organ donation health professionals elect to attend, and the College of Intensive Care Medicine made completion of the core module a mandatory training requirement for intensive care trainees from 2014 [41]. However, role-play alone may not adequately replicate the emotional nature of donation conversations.

In NSW, training for health professionals selected as “designated requesters” to lead donation conversations has been supplemented by a simulation program [4]. Piloted in 2012 the program uses real donation scenarios with standardised relatives played by professional actors [42]. Health professionals are able to rehearse, review and reflect on their developing effective communication skills when offering donation in a protected learning environment, and thereby become more comfortable discussing these topics. These ‘best practice’ methods involving use of specialised requesters to lead deceased organ donation discussions with families are based on work from other countries adapted to but not formally tested in Australian conditions. This study will examine implementation of a ‘best practice’ family approach intervention and identify its effectiveness in terms of family consent rates, and later decisional satisfaction or regret.

## Methods/Design

### Aims and hypothesis

The aim of this study is to examine the process of organ donation decision-making, and to test whether changes in requesting practices change rates of family consent for organ donation and other family-based outcomes. A secondary aim is to examine whether changes in requesting practices result in increased satisfaction by families with their donation decision. The hypothesis for the trial component is that, compared to current usual practice, a ‘best practice’ family approach intervention will increase the family consent rate for deceased organ donation.

### Design

This is a pre-post intervention design where rates of consent for organ donation for at least six months before implementation of the program in each ICU will be compared to the rates of consent for organ donation under the intervention.

### Settings

The study will be conducted in the ICUs or locations such as Emergency Departments when the patient is managed by ICU health professionals, of nine metropolitan and rural hospitals in NSW, with follow-up conducted by the NSW OTDS.

### The COMmunication with families regarding ORgan and tissue donation after death in intensive care (COMFORT) intervention

The intervention is a modification of current standard practice procedures for offering donation to families of potential organ donors. There are six essential ‘best practice’ elements of the intervention:

1. A designated requester has primary responsibility for discussions regarding organ donation with the family of a potential donor. Primary communication with families regarding end of life management and death remains the responsibility of the managing team.
2. Designated requesters are volunteer intensivists, experienced critical care nurses, or social workers who have been deemed appropriate by the site principal investigator/ICU department head to undertake the role, and complete mandatory training (see Additional file 1). Up to six designated requesters are estimated to be required at each study hospital.
3. The offer of donation is separated from the conversation where families are informed of the patient’s death. It is important the family have accepted the inevitability of death before donation is raised [25].
4. If families raise the topic of organ or tissue donation, the managing health professional sensitively defers the first donation conversation to the designated requester.
5. Donation conversations are conducted within the structure of a family meeting, based on evidence-based guidelines for high quality communication regarding end-of-life care [23, 28–30].
6. The requester uses a ‘balanced approach’: information is provided in a proactive manner, using open-ended questions to encourage active participation of family members in discussion. Information is provided about the benefits of organ and tissue donation for both families and recipients [39].

Key features to which the multidisciplinary team and designated requesters are expected to adhere are set out in Table 1.

**Table 1:**
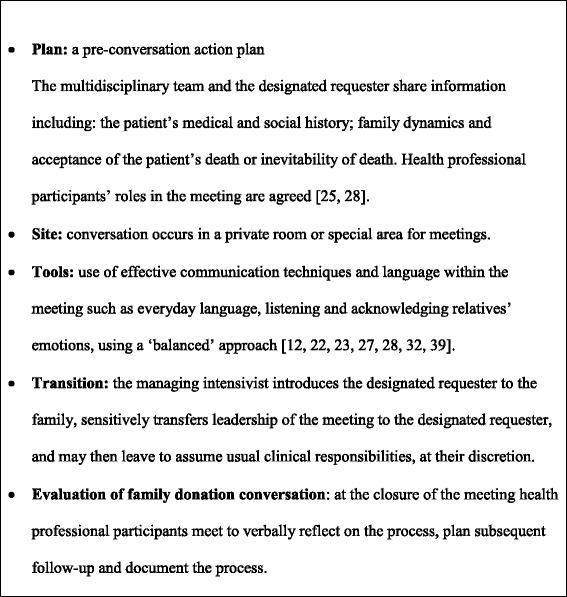
Key adherence criteria for delivery of the ‘COMFORT’ intervention

The training provides designated requesters preparation and opportunities to practice elements three to six of the COMFORT intervention. Designated requester training requirements are completed subsequent to attending the Organ and Tissue Authority FDC core and practical modules and the NSW simulation workshop (see Additional file 1) [40, 42]. Subsequent attendance at the simulation workshop is required for annual refresher training for DSNs and social workers, and 18 monthly for intensivists, for the duration of the COMFORT study.

As the study intervention is a modification to health service delivery, it is led in each hospital by local specialist donation nurses and doctors. Education sessions are delivered as required to colleagues in the departments of emergency medicine, intensive care, neurosurgery and social work to support and provide information and feedback on the implementation process of the new intervention. Information is collected from and on the health professionals involved in each organ donation event to track intervention fidelity.

### Participants

Participants in this study are the families of patients who are potential deceased organ and tissue donors and the health professionals involved in each organ donation event. Members of the managing team identify a possible deceased organ donor who is apparently medically suitable for organ donation, and notify the donation specialist at the hospital or the OTDS. To be eligible for the study a donation event must meet all inclusion and no exclusion criteria as detailed below:

#### Inclusion criteria

Donation events identified by patients of all ages managed in the ICU or under the care of ICU health professionals, who are potential deceased organ and tissue donors. For the primary endpoint only, patients must not have registered their donation wishes.

#### Exclusion criteria

Donation events or patients who fulfil one or more of the following criteria:

- A patient who is not medically suitable for deceased organ and tissue donation;
- A patient who does not have a SaNOK to participate in donation conversations;
- An adult patient in the ICU who is able to provide first person consent for deceased donation, for example a patient with cervical spine injury;
- A patient who is suitable to donate only tissue after death.

### Endpoints

The primary endpoint for the study is the family consent rate for deceased organ donation where the potential donor had not previously registered their donation wishes.

Secondary endpoints are: health professionals’ adherence rates to core elements of the COMFORT intervention; identification of predictors of family donation decision; and the proportion of SaNOK who report they regretted their final donation decision at around 90 days after enrolment.

### Study outline

**Fig. 1:**
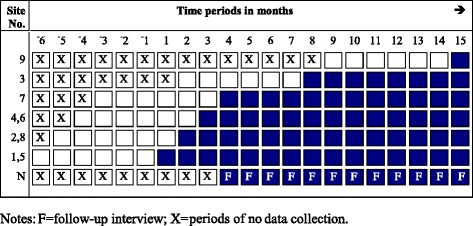
Chart of the study design and data collection periods. Shaded cells represent intervention periods; Blank cells represent control periods. Each cell represents a data collection point, except those with ‘X’ *N =* NSW Organ and Tissue Donation Service, f = follow-up interviews

In line with usual practice, potential donors are identified, a registry check is performed to find any recorded preference regarding organ donation by the patient, and the process of assessing medical suitability is commenced (not necessarily in that order). In the COMFORT intervention condition the managing team is responsible for delivering the news of death, and contacts the donation specialist/designated requester to plan the approach to the family and initiate the donation offer (as above and Table 1). In the pre-intervention control condition, the donation conversation is managed by the healthcare professional(s) and processes are the usual practice in that setting. A chart of the study design and data collection periods is shown in Fig. 1. The site initiation visit is the point where hospitals crossover from the control to the intervention condition. In both groups families may take up to 72 h for a final donation decision.

Bereavement support provided by the OTDS Family Support Coordinator is offered to families who participated in the donation requesting conversation regardless of their final organ donation decision. Senior next of kin, or the delegated decision maker, who agrees to bereavement aftercare is invited to participate in the subsequent follow-up interview at day 90 after enrolment.

### Evaluation: data collection and management

#### Setting

To characterise and describe the setting, data recorded at the beginning and at completion of the study at each site include: number of hospital and ICU beds; categories (medical/surgical/paediatric) of patients admitted to the ICU; areas of specialty; medical, nursing and allied health staffing establishments and ratios; availability of private meeting rooms; frequency of multidisciplinary communication (ward rounds; and family meetings).

#### Current control

To describe practice for the ‘control condition’ i.e. pre-intervention details of donation events for the period of six months before each site implements the intervention and joins the program will be extracted retrospectively. Data will include: eligibility for deceased organ donation; patients’ date of birth, donation intent registered on their motor vehicle licence and the Australian Organ Donor Register; initiator of the donation conversation; family final donation decision, and outcome of the donation event.

#### Intervention period screening

A screening log is maintained at the OTDS of notification of patients who are apparently medically suitable for deceased organ donation during the study intervention period. DSNs routinely coordinate data collection at each hospital and forward completed forms to the OTDS for validation and consistency checks.

#### Donation events

A unique number is allocated to each potential organ donor at enrolment. Data from eligible potential donation events include donation pathway (i.e. brain or circulatory death), designation of initiator of the family donation conversation, donation intent on a register, and family donation decision. Also characteristics of potential donors such as: date of birth, gender, ethnicity, religion, primary event/cause of death, dates and times of ICU admission and death, commencement of retrieval surgery, and family contact details.

#### Family donation conversation

Adherence to elements of the family donation conversation is consecutively recorded on a case report form for each donation event by self-report from the observations of health professionals who participated in the meeting. Details include general features of a structured family meeting and specific topics discussed in the first meeting. The role of the ICU health professional who led the initial family donation conversation (termed the ‘requester’) is central to this process; their demographic, training and number of donation requesting conversations experiences in the preceding calendar year are collected by self report. Reporting is undertaken at the closure of the conversation to minimise potential recall bias. A consensus approach is used with equal weight given to each health professional’s observations. Completion of the case report form may extend to one week. The final donation decision by the family is recorded at conclusion of the process. Reasons stated by the family and/or perceived by the health professional for that decision at that time point will be noted. See Additional file 2 for the case report form.

#### Follow-up with the SaNOK

Follow-up bereavement aftercare will be offered to SaNOK as part of the donation conversation process. An invitation to participate in the follow-up interview and the participant information sheet and consent form are posted to SaNOK who agreed to follow-up approximately two weeks before the 90-day post bereavement time point. Written or verbal consent are subsequently sought from the SaNOK for the follow-up interview and to audio-record it, although the interviewer will take notes rather than audio-recording if participants prefer. This contact procedure is similar to that used in previous research with families of potential organ donors [43]. Hence the three-stage process for consent to follow-up entails:

1. Initial verbal consent to the offer of bereavement aftercare, followed by
2. Provision of written information and written or verbal consent, and
3. Confirmation of consent before conducting the interview, and for audio-recording.

The OTDS Family Support Coordinator conducts the telephone interviews with senior next of kin who agree to bereavement aftercare. Data sought include: demographic details; bereavement support received; information received regarding organ and tissue donation, and family members’ perception as to whether this was adequate for them to make a decision; previous discussions with their relative regarding organ and tissue donation; if they would now make the same donation decision, and their decision rationale.

#### Consent rates over time

To describe trends in family donation decisions over the same time period as the COMFORT study and identify any trends/changes over time notification data from all NSW hospitals ICUs and Emergency Departments are collected. Data variables collected have been listed at *Donation events*, above (with the addition of ethnicity, religion, ICU stay and retrieval data as appropriate).

### Statistical methods

The sample size calculation was performed using the Simon’s two-stage design [44]. 140 eligible next of kin families are required to be approached to provide consent for organ donation. This will yield 80% power with 95% confidence to exclude a consent rate of 29% in favour of a clinically worthwhile rate of 40% for the intervention. An eligible next of kin family are those of patients who had not registered their donation decision.

Additionally, in the first 46 eligible next of kin families who are approached, if less than 15 have consented to organ donation, consideration will be given to modifying the study.

### Statistical analysis

A patient flow-chart shows the number of patients eligible and the numbers enrolled at each site. No imputation is envisaged to be performed for next of kin where the primary outcome is unknown. Summary statistics will be presented for continuous variables, and counts and percentages presented for categorical variables.

All analyses will apply the intention to treat principle. For example in a case where the intervention was not properly followed, such as the SaNOK had organ donation raised by an inappropriate requester instead of a designated requester, the patient will still be included in the study and considered to have received the intervention.

Paediatric cases defined as those aged less than or equal to 16 years, will be analysed as a separate cohort.

#### To address the primary endpoint

- Consent rates provided by the next of kin for organ and tissue donation where the potential donor had not registered their decision will be calculated.

#### To address the secondary endpoints

1. Adherence to core elements of the intervention will be obtained via the case report form and rates calculated.
2. The proportion of all next of kin who report they regretted their final decision either to consent or to decline donation at 90 days will be calculated with 95% Confidence Interval.
3. Characteristics of the donation process including staff adherence to core elements of the intervention and demographic characteristics of the potential donor, senior next of kin and of the requester will be explored. Exploratory analysis using both univariate and multivariate regression methods will be used as needed. A *p-*value of < 0.05 will be considered statistically significant for retention in the multivariate model and only univariate variables with a *p-*value <0.20 will be considered for inclusion to the multivariate model.

Categorical data (e.g. details of gender, religion, ethnicity of patient and health care professional and reasons to consent or decline donation) will be summarised by frequencies and percentages. Continuous data (e.g. age, time in family meetings) will be summarised using the mean and standard deviation.

#### Additional analysis: consent rates over time

Consent rates for hospitals that have participated in the “best practice” family approach intervention training will be compared up to six months before the site initiation visit with the consent rates under the intervention.

To establish the baseline (pre COMFORT) and concurrent data trends NSW state-wide, consent rates in hospitals not participating in “best practice” family approach intervention training at any point during the study will be presented over time.

The consent rates between NSW hospitals that had ICU health professionals trained in the intervention and NSW hospitals that never received the training will be presented. This analysis will only include families that were approached about organ donation before the hospital introducing the intervention training.

#### Additional analysis: follow-up data

Summary statistics will be provided showing the next of kin’s knowledge of their loved one’s organ donation wishes.

Summary statistics of the next of kin’s demographic data and their circumstances of the organ donation request will be presented by donation decision and by the next of kin’s enduring (90 days) regret or support of their donation decision.

### Monitoring

The project manager will conduct a site initiation visit and subsequent visits to each study site during the intervention phase to support protocol compliance and adherence to good clinical practice in research. Hospital records, source documents and other study files will be accessible at all study sites for monitoring and auditing purposes. The OTDS will regularly monitor recruitment by screening notifications of possible organ donors to the organisation.

### Ethical considerations

The study is a pragmatic evaluation “in practice” of adoption of key elements of the FDC training representing adherence to evidence-based guidelines for end-of-life communication with families. As part of routine care, health professionals delivering this intervention are able to access existing psychological supervision for support should they wish.

It is possible that contacting families may cause them anxiety or distress. This possibility is addressed by offering bereavement aftercare provided by the OTDS Family Support Coordinator to families who declined organ donation, currently not available to them under standard care conditions, but routinely offered to families who agreed to donation. In light of their existing relationship with the family, their counselling expertise and independence from the managing clinicians, the OTDS Family Support Coordinator will conduct the Day-90 interview subsequent to family verbal or written consent. Participants are able to change their mind at any time without affecting eligibility for ongoing bereavement support.

## Discussion

This study has been designed to evaluate the uptake and outcomes of a ‘best practice’ intervention entailing a framework of evidence-based family conversations led by a skilled ‘designated requester’ convened after the news of a loved one’s death has been delivered, to make decisions regarding end-of-life care and organ donation. This is an important initiative to identify ‘what works’ in usual clinical settings when requesting organ donation in critical care environments, both in terms of what changes in practice healthcare professionals are willing and able to adopt, and what effect this may have on desired outcomes.

A strength of this study is its pragmatic, ‘real world’ nature; findings will be immediately relevant and potentially generalisable to other clinical settings as the study is conducted as part of routine care. Standard care procedures of the ‘control’ condition will be detailed, enabling other sites to make comparisons between their practice and the practice employed in both ‘conditions’ of this study. Introduction of the designated requester role to lead the initial family donation conversation will be examined in both metropolitan and rural ICUs. Reasons for health professionals’ decisions to deviate from the intervention pathway will be collected prospectively, to maximise understanding of the results of this study and identify procedures to review or to incorporate in future innovative implementation models.

Further strengths include characterisation of the donation requesting process in such a way as to enable identification of features of ‘best practice’ that are important both from Australian healthcare professionals’ and families’ perspectives, particularly in cases when they were unaware of the donation preference of the potential organ donor. Reasons for the families’ donation decisions are recorded contemporaneously, thereby minimising the effect of recall bias. Use of an interviewer independent of the hospital managing team for family follow-up interviews is intended to facilitate open disclosure of their experiences of events.

There are some limitations of this study. Firstly, there were constraints on design. A pre-post intervention design was chosen in order to maximise recruitment and obtain an adequate sample size within a reasonable time period. Alternative designs were not feasible. For example, a randomised controlled trial design would have incurred high likelihood of contamination of ‘control’ sites by features of ‘best practice’ once national education began to be delivered in NSW. Cluster randomised control designs were discounted due to insufficient numbers of hospitals in NSW. A stepped wedge design was not possible as the crossover point for each site was primarily dependent upon staff release for the designated requester training, which in turn was dependent on local staffing; consequently this could not be randomly allocated. The pre-post design enabled all units to participate in the intervention and made economical use of ‘control’ data from every site.

Secondly, there are some potential threats to the validity of study findings. The delivery of the national FDC training may result in increased family consent rates independent of the study intervention. However, this training is only one part of the support planned for core elements of this intervention, so, if effective, consent rates would still be expected to increase more rapidly under the ‘intervention’ compared to the ‘control’ condition. We are unable to gauge the impact of ongoing community education activities directed to increase the proportion of people who register their donation decision. Selection bias is acknowledged for the follow-up interviews, in that those families who either were not offered or do not wish to have bereavement aftercare are excluded from this portion of the study.

Practical issues related to the delivery of a multi-centre trial made a staged roll-out of implementation necessary. However, operational issues, including the turn-over of DSNs and the time required for the requisite numbers of designated requesters to complete all training workshops (up to a year), caused delay in recruitment at some sites. The original plan for sequential start in equally spaced time periods could not occur. Practical issues may also affect protocol adherence; for example, without funding to allocate designated requesters to on-call rosters, availability is dependent on usual rostering procedures. Implementation of the intervention is therefore pragmatic being dependent on factors that will equally affect any future delivery as part of routine practice as well as a research intervention.

## Additional files

Additional file 1: Summary of training program. (DOCX 115 kb)
Additional file 2: COMFORT study case report forms 2–6. (PDF 264 kb)

## Abbreviations

DSN: Donation specialist nurse
FDC: Family donation conversation
HREC: Human research ethics committee
ICU: Intensive care unit
NSW: New South Wales
OTA: Organ and Tissue Authority
OTDS: Organ and Tissue Donation Service
SaNOK: Senior available next of kin
UTS: University of Technology Sydney

## References

[1] Dominguez-Gil B, Delmonico FL, Shaheen FAM, Matesanz R, O’Connor K, Minina M (2011). The critical pathway for deceased donation: reportable uniformity in the approach to deceased donation. Transpl Int.

[2] International Registry in Organ Donation and Transplantation (IRODaT). Database. 2011. http://www.irodat.org. Accessed 5 Oct 2015.

[3] Human Tissue Act, 1983, Stat. Act no. 164. 1983 (1983).

[4] NSW Ministry of Health. Increasing Organ Donation in NSW – Government Plan 2012. http://www.health.nsw.gov.au/organdonation/Pages/increasing-organ-donation.aspx. Accessed 20 Dec 2016.

[5] Organ and Tissue Authority. Donation and Transplantation Performance Report for 2011. http://www.donatelife.gov.au/sites/default/files/files/performancereportupdate2011.pdf. Accessed 20 Sept 2014.

[6] Organ and Tissue Authority. Donation and Transplantation Performance Report for 2013. http://www.donatelife.gov.au/sites/default/files/files/OTA_2013_Performance_Report.pdf. Accessed 20 Dec 2016.

[7] NHS Blood and Transplant. Consent Authorisation. http://www.odt.nhs.uk/donation/deceased-donation/consent-authorisation/. Accessed 10 Dec 2014.

[8] Organ and Tissue Authority. Facts and Statistics. http://www.donatelife.gov.au/discover/facts-and-statistics. Accessed 5 Oct 2015.

[9] Irving MJ, Tong A, Jan S, Cass A, Chadban S, Allen RD, Craig JC, Wong G, Howard K (2012). Community attitudes to deceased organ donation: a focus group study. Transplantation.

[10] Matesanz R, Dominguez-Gil B, Coll E, de la Rosa G, Marazuela R (2011). Spanish experience as a leading country: what kind of measures were taken?. Transpl Int.

[11] Vincent A, Logan L (2012). Consent for organ donation. Br J Anaesth.

[12] Curtis JR, Engelberg RA, Wenrich MD, Shannon SE, Treece PD, Rubenfeld GD (2005). Missed opportunities during family conferences about end-of-life care in the intensive care unit. Am J Resp Crit Care Med.

[13] McDonagh JR, Elliott TB, Engelberg RA, Treece PD, Shannon SE, Rubenfeld GD, Patrick DL, Curtis JR (2004). Family satisfaction with family conferences about end-of-life care in the intensive care unit: increased proportion of family speech is associated with increased satisfaction. Crit Care Med.

[14] Williams M, Lipsett P, Rushton C, Grochowski E, Berkowitz I, Mann S, Shatzer J, Short M, Genel M (2003). The physician’s role in discussing organ donation with families. Crit Care Med.

[15] Organ and Tissue Authority. The Australasian Donor Awareness Program (ADAPT). http://www.donatelife.gov.au/idat. Accessed 20 Dec 2016.

[16] Mullins GC, Simes D, Yuen KJ (2012). Approaching families for organ donation-intensivists’ perspectives. Anaesth Int Care.

[17] Pearson IY, Zurynski Y (1995). A survey of personal and professional attitudes of intensivists to organ donation and transplantation. Anaesth Int Care.

[18] Thomas SL, Milnes S, Komesaroff PA (2009). Understanding organ donation in the collaborative era: a qualitative study of staff and family experiences. Int Med J.

[19] Sque M, Long T, Payne S (2005). Organ donation: key factors influencing families’ decision-making. Transplant Proc.

[20] Simpkin AL, Robertson LC, Barber VS, Young JD (2009). Modifiable factors influencing relatives’ decision to offer organ donation: systematic review. BMJ.

[21] Shafer TJ, Ehrle RN, Davis KD, Durand RE, Holtzman SM, Van Buren CT, Crafts NJ, Decker PJ (2004). Increasing organ recovery from level I trauma centers: the in-house coordinator intervention. Prog Transplant.

[22] Siminoff LA, Marshall HM, Dumenci L, Bowen G, Swaminathan A, Gordon N (2009). Communicating effectively about donation: an educational intervention to increase consent to donation. Prog Transplant.

[23] Australian and New Zealand Intensive Care Society. The ANZICS Statement on Death and Organ Donation. (Edition 3.2). Melbourne: ANZICS; 2013. http://www.anzics.com.au/Pages/DaOD.aspx. Accessed 21 Aug 2015.

[24] NHS National Institute for Health and Clinical Excellence. Organ donation for transplantation: improving donor identification and consent rates for deceased organ donation. National Institute for Health and Clinical Excellence, Manchester, UK. 2011. http://www.nice.org.uk/guidance/CG135. Accessed 28 Apr 2013. 23534085

[25] Consent/Authorisation Best Practice Development Group. Approaching the families of potential organ donors: best practice guidance. NHS Blood and Transplant, UK. 2015. http://www.odt.nhs.uk/pdf/family_approach_best_practice_guide.pdf. Accessed 5 Jan 2016.

[26] Siminoff LA, Gordon N, Hewlett J, Arnold RM (2001). Factors influencing families’ consent for donation of solid organs for transplantation. JAMA.

[27] Billings JA (2011). The end-of-life family meeting in intensive care part II: family-centered decision making. J Pall Med.

[28] Billings JA, Block SD (2011). The end-of-life family meeting in intensive care part III: a guide for structured discussions. J Pall Med.

[29] Curtis JR, White DB (2008). Practical guidance for evidence-based ICU family conferences. Chest.

[30] Lautrette A, Ciroldi M, Ksibi H, Azoulay E (2006). End-of-life family conferences: rooted in the evidence. Crit Care Med.

[31] Billings JA (2011). The end-of-life family meeting in intensive care part I: indications, outcomes, and family needs. J Pall Med.

[32] Lautrette A, Darmon M, Megarbane B, Joly LM, Chevret S, Adrie C (2007). A communication strategy and brochure for relatives of patients dying in the ICU. N Engl J Med.

[33] Luskin RS, Glazier AK, Delmonico FL (2008). Organ donation and dual advocacy. N Engl J Med.

[34] Zink S, Wertlieb S (2006). A study of the presumptive approach to consent for organ donation: a new solution to an old problem. Crit Care Nurs.

[35] Siminoff LA, Agyemang AA, Traino HM (2013). Consent to organ donation: a review. Prog Transplant.

[36] Jacoby L, Jaccard J (2010). Perceived support among families deciding about organ donation for their loved ones: donor vs nondonor next of kin. Am J Crit Care.

[37] Rodrigue JR, Cornell DL, Howard RJ (2008). The instability of organ donation decisions by next-of-kin and factors that predict it. Am J Transplant.

[38] Organ &Tissue Authority. National Reform Programme. http://www.donatelife.gov.au/about-us/national-reform-programme. Accessed 14 Sept 2014.

[39] Mulvania P, Mehakovic E, Wise C, Cass Y, Daly TA, Nathan HM (2014). Successful international collaboration improves family donation conversations resulting in increased organ donation. Transplant Proc.

[40] Organ and Tissue Authority. Professional Education Package: Family Donation Conversation. http://www.donatelife.gov.au/professional-education-package. Accessed 21 Aug 2015.

[41] Organ and Tissue Authority. Family Donation Conversation Core Module. http://www.donatelife.gov.au/family-donation-conversation-core-module. Accessed 21 Aug 2015.

[42] Potter JE, Gatward JJ, Kelly MA, McKay L, McCann E, Elliott RM, Perry L. Simulation-based communication skills training for experienced clinicians to improve family conversations about organ and tissue donation. Prog Transplant. In press. 10.1177/1526924817731881 29187126

[43] Neate SL, Marck CH, Skinner M, Dwyer B, McGain F, Weiland TJ, Hickey BB, Jelinek GA (2015). Understanding Australian families’ organ donation decisions. Anaesth Int Care.

[44] Simon R (1989). Optimal two-stage designs for phase II clinical trials. Cont Clin Trials.

