# Supplementary material for: COMmunication with Families regarding ORgan and Tissue donation after death in intensive care (COMFORT): protocol for an intervention study
Source: BMC Health Serv Res. 2017 Jan 17;17:42. doi: 10.1186/s12913-016-1964-7 (PMC5240419; doi:10.1186/s12913-016-1964-7)
Supplement: Additional file 1: — Summary of training program. (DOCX 115 kb) [file 12913_2016_1964_MOESM1_ESM.docx]

# Summary of training program

The specialised communication training for designated requesters has two components: the national Family Donation Conversation (FDC): core and practical modules and the New South Wales (NSW) Designated Requester Simulation Training Program [1, 2].

## Family Donation Conversation: core module

This is a two-day workshop on the advanced theory behind family donation conversations, and introduces specialised communication tools for requesters to support potential donor families in acute grief while raising organ and tissue donation. Training is provided on the new Australian model, the “balanced approach” of offering organ donation to families to ensure their donation decision is based on information, is proactive, and would be repeated if asked at a future time. Attendance is capped at 30 health professionals with two facilitators each workshop [3].

From February 2013 workshops are facilitated from a national group of Australian health professionals, known as the LEAD (Learn, Evolve, Achieve, Discover) trainers. These people are intensivists, experienced donation nurses, and grief and family support specialists, who had completed a training program provided by the Gift of Life Institute, Philadelphia [3].

Revised in 2014, aims of the core workshop are to provide participants with an understanding of:

- Specific elements of family care and communication.
- Range of reactions experienced by families who receive catastrophic news.
- Key factors in the process, timing and sequence of the donation conversation.
- Best practices for supporting families in grief and in the donation conversation.
- Strategies to ensure informed and enduring decision-making regarding donation [4].

## Family Donation Conversation: practical module

This is a one-day practical workshop for health professionals who have completed the FDC: core module, to practice planning and to manage a complex family donation conversation. Three experienced donation specialists, including LEAD trainers, facilitate workshops.

Revised in 2014, aims of the practical module are to enable participants to:

- Reflect on learnings from the core module.
- Identify and discuss the benefits of separating the conversations about death and donation.
- Prepare and plan a family donation conversation using a team approach.
- Practise the first conversation of raising donation with a family.
- Practise responses to family concerns.
- Use tools to support learning and practice [5].

## Designated Requester Simulation Training Program

This accredited training was developed in partnership with the NSW Organ and Tissue Donation Service and the University of Technology Sydney, and builds on communication skills from previous workshops. These half-day workshops are conducted in university simulation laboratories in small groups with professional actors, with a maximum of four participants. Real donation scenarios are used with standardised relatives played by professional actors. Donation scenarios include pathways of donation after brain death and donation after circulatory death determination. The role of the designated requester and the family donation conversation are highlighted. Debriefing is led by qualified experts with video assisted reflective debriefing as a key tool in training evaluation, in addition to feedback from the actors both ‘in’ and ‘out-of-character’ [2].

## References

1. Organ and Tissue Authority. Professional Education Package: Family Donation Conversation. 2015. http://www.donatelife.gov.au/professional-education-package. Accessed 21 Aug 2015.

2. McCann E, Gatward J. Introducing simulation training to improve the organ donation conversation. The 12th Congress of the International Society for Organ Donation and Procurement; November 21-24; Sydney, Australia: Transplantation; 2013. p. S237.

3. Mulvania P, Mehakovic E, Wise C, Cass Y, Daly TA, Nathan HM. Successful international collaboration improves family donation conversations resulting in increased organ donation. Transplant Proc. 2014;46(6):2058-65.

4. Organ and Tissue Authority. Family Donation Conversation Core Module. 2014. http://www.donatelife.gov.au/family-donation-conversation-core-module. Accessed 21 August 2015.

5. Organ &Tissue Authority. Family Donation Conversation Practical Module. 2014. http://www.donatelife.gov.au/family-donation-conversation-practical-module. Accessed 21 August 2015.
