# Supplementary material for: COMmunication with Families regarding ORgan and Tissue donation after death in intensive care (COMFORT): protocol for an intervention study
Source: BMC Health Serv Res. 2017 Jan 17;17:42. doi: 10.1186/s12913-016-1964-7 (PMC5240419; doi:10.1186/s12913-016-1964-7)
Supplement: Additional file 2: — COMFORT study case report forms 2–6. (PDF 264 kb) [file 12913_2016_1964_MOESM2_ESM.pdf]

Form 2: Pre-conversation action plan

2.1 **Date:** |__| |__| / |__| |__| / |_2_| |_0_| |_1_| |__| *(dd/mm/yyyy)*

Start time:|__| |__|:|__| |__| 24 hr

2.2 **Participants: health professionals** — Please tick ☑ one per person

- ☐ MD: Intensivist (not managing the patient)
- ☐ MD: Intensivist (managing the patient)
- ☐ RN: Donation Specialist Nurse
- ☐ MD: Registrar (ICU)
- ☐ RN: allocated care of patient today
- ☐ MD: Resident (ICU)
- ☐ RN: other_______________________________
- ☐ SW: Social Worker
- ☐ HC: Hospital chaplain
- ☐ Other, *specify*:___________________________
- ☐ Other spiritual support: ___________________
- ☐ Interpreter, language:_____________________
- ☐ Designated requester (if not working in designation above)

2.3 **Who will lead the conversation today** — Please tick ☑ one

- ☐ RN: Donation Specialist Nurse
- ☐ MD: Intensivist (managing the patient)
- ☐ Other, *specify*:
- ☐ Designated requester

2.4 **News of brain death or inevitability of death delivered to the family**

Date: |__| |__| / |__| |__| / |_2_| |_0_| |_1_| |__| *(dd/mm/yyyy)* **Time**:|__| |__|:|__| |__| 24 hr

2.5 **Was organ donation raised with the family before this planning meeting?**

- ☐ Yes, the family raised it
- ☐ Yes, the managing team raised it
- ☐ No

2.6 **If yes to 2.5, who discussed organ donation with them?**

- ☐ Designation:____________________________

2.7 **Topics of planning discussion** — Please tick ☑ all that apply

- ☐ Planned date and time of the donation conversation?
- ☐ Goals of this conversation?
- ☐ Who should be present from the managing team?
- ☐ Roles for participants in the donation conversation?
- ☐ Identity of the lead decision maker/senior next of kin?
- ☐ The patient's medical history and events in the hospital?
- ☐ Status of next of kin in acceptance of death or inevitability of death?
- ☐ Everyone here who should be for the next of kin?
- ☐ Any conflict experienced within the family?
- ☐ Any conflict experienced between family and treating team?
- ☐ Current emotional capacity of the next of kin i.e level of coping?
- ☐ Next of kin's questions about medical treatment clearly answered?
- ☐ Next of kin's physical needs attended (food, fluid, hygiene)?
- ☐ Other available support for next of kin?
- ☐ Other, *specify*:

2.8 ☐ **Pre-conversation action plan did not occur**

2.9 **Donation Specialist Nurse introduced to the family**

Date: |__| |__| / |__| |__| / |_2_| |_0_| |_1_| |__| *(dd/mm/yyyy)* **Time**:|__| |__|:|__| |__| 24 hr

Form 3: Meeting for the family donation conversation

Part A: First meeting *(to be completed with health professionals who attended this meeting)*

3.1 **Date:** |__| |__| / |__| |__| / |_2_| |_0_| |_1_| |__| *(dd/mm/yyyy)*

Start time:|__| |__|:|__| |__| 24 hr **Stop time:**|__| |__|:|__| |__| 24 hr

3.2 **Location** Please tick ☑ one

☐ Patient's bedside ☐ Private room set aside for meetings ☐ Other, *specify*:_______________

3.3 **Who led the conversation** Please tick ☑ one (& complete Form 5)

☐ RN: Donation Specialist Nurse ☐ MD: Intensivist (managing the patient)
☐ Other, *specify*: ☐ Designated requester

3.4 **Transparent introduction of the requester (by the managing team)** Please tick ☑ one

☐ Yes, stated works in organ donation ☐ NA, designated requester not introduced
☐ No, blinded with role stated in general terms ☐ NA, one of the managing team led the meeting

3. 5 **Time the managing intensivist left before the meeting closed** Complete one of Y, N or NA

3.5.1 **Yes**, left the meeting at time: |__| |__|:|__| |__|24 hr

3.5.2 **No**, did not leave because: Please tick ☑ the main reason for "N"

☐ Also a designated requester (DR)
☐ Led the meeting because a DR was unavailable
☐ Stayed to answer clinical management questions
☐ Stayed to observe the method of communication or to mentor a DR
☐ *Specify other reason for staying*:

3.5.3 ☐ **NA** the managing intensivist did not attend this meeting.

3.5.4 *If applicable*: the DR left before the meeting closed at time: |__| |__|:|__| |__|24 hr

3.6 **Topics discussed** Please tick ☑ all that apply and circle "F" if raised by a member of the family

☐ F Understanding of brain death
☐ F Understanding of plan to withdraw/withhold treatment
☐ F Discussion about loved one, circumstances of death etc
☐ F Rare opportunity for organ donation
☐ F Emphasis on the benefits of donation and the potential to help others
☐ F Description of the organ donation process
☐ F Does not incur additional costs to family
☐ F Knowledge of patient's donation wishes
☐ F Reassurance regarding the fairness of organ allocation
☐ F Other, *specify:*

3.7 **Participants: health professionals** Please tick ☑ one per person (more on page 2)

☐ MD: Intensivist (not managing the patient) ☐ MD: Intensivist (managing the patient)
☐ RN: Donation Specialist Nurse ☐ MD: Registrar (ICU)
☐ RN: allocated care of patient today ☐ MD: Resident (ICU)
☐ RN: other___________________________ ☐ SW: Social Worker
☐ HC: Hospital chaplain ☐ Other spiritual support:_____________________

Form 3: Meeting for the family donation conversation

☐ Other, *specify*:________________________ ☐ Interpreter, language:________________________
☐ Designated requester (if not working in designation above)

3.8 Participants: family — **Please tick ☑ all that apply and write number of attendees**

☐ Spouse/partner/de facto/same sex partner (include ex) No.: |__| |__|
☐ Adult child (18yrs or older) (include step children) No.: |__| |__|
☐ Parent (include step or adoptive parents) No.: |__| |__|
☐ Adult sibling No.: |__| |__|
☐ Adult sibling's partner No.: |__| |__|
☐ Grandparent No.: |__| |__|
☐ Other, *specify*: No.: |__| |__|

3.9 Designation of the SaNOK, *specify relationship to the potential donor*:

3.10 *If applicable*: The SaNOK **delegated decision making** to *(specify relationship to the potential donor)*:

3.11 Outcome of the initial family donation conversation — **Please tick ☑ one**

☐ Definite in principle consent ☐ Initial decline: "reactive no"
☐ Agreement to consider ☐ Definite in principle decline
☐ Other, *specify*:

Part B: Final outcome *(to be completed with the individual who led the first conversation)*

3.12 Final donation decision — **Please tick ☑ one (and complete Form 4)**

☐ Written consent ☐ Definite in principle decline
☐ Other, *specify*:

3.13 Date: |__| |__| / |__| |__| / |_2_| |_0_| |_1_| |__| *(dd/mm/yyyy)* **Time**:|__| |__|:|__| |__| 24 hr

3.14 Total of family donation conversations to reach the final donation decision: No.: |__| |__|

3.15 Did each family member who attended the initial donation conversation attend all follow up meetings?

☐ Yes ☐ No ☐ NA (only one meeting)

3.16 Please comment if response was "No" in 3.15:

3.17 Procurement surgery commenced incision time *(if applicable)*

Date: |__| |__| / |__| |__| / |_2_| |_0_| |_1_| |__| *(dd/mm/yyyy)* **Time**:|__| |__|:|__| |__| 24 hr

3.18 Revocation of consent at the hospital *(if applicable)* **(and complete Q 3.19)**

Date: |__| |__| / |__| |__| / |_2_| |_0_| |_1_| |__| *(dd/mm/yyyy)* **Time**:|__| |__|:|__| |__| 24 hr

3.19 Please comment on reason(s) for revocation of consent stated by SaNOK.

Reason(s) for the final donation decision

Categorise reasons as:
S= **stated** verbally by the senior available next of kin to the requester and/or delegate
P= **perceived** by the requester

4.1: Reasons for consent *(selected after completing Q3.12)*

Circle S or P

S	P	What other donor families have shared
S	P	Knew donor’s wishes from donor registry / driver’s licence
S	P	Knew donor’s wishes from previous discussion
S	P	Enabling someone else to live a better life
S	P	Donor would have wanted to help others
S	P	Opportunity for something positive to come out of a tragedy
S	P	Part of a relative living on in someone else
S	P	Previous personal experience with donation
S	P	The donor had never said “no”
S	P	Other, *specify*:

4.2: Reasons for decline *(selected after completing Q 3.12)*

Circle S or P

S	P	Concerns over delay to funeral/burial process
S	P	Concerns regarding integrity of process e.g unfair organ allocation, organ selling
S	P	Disagreements among the family group
S	P	Dissatisfaction with the patient’s treatment in the ICU
S	P	Dissatisfaction with the patient’s treatment in other areas of the hospital
S	P	Dissatisfaction with duration of the donation process
S	P	Longstanding negative views on organ donation
S	P	Not wishing surgery to the body/concerns regarding disfigurement
S	P	Emotional exhaustion
S	P	Religious/cultural reasons
S	P	Decided on their own that organs would not be suitable
S	P	Thought that the patient had suffered enough
S	P	Unable to accept death, lack of understanding of brain death
S	P	Uncertainty regarding the patient’s wishes
S	P	Knew donor’s wishes from donor registry / driver’s licence
S	P	Knew donor’s wishes from previous discussion
S	P	Other*, specify*:

Form 5: Requester details *(to be completed by the individual who led the first meeting in Form 3)*

5.1 Requester details

5.1.1 Date of birth: |__| |__| / |__| |__| / |_1_| |_9_| |__| |__| *(dd/mm/yyyy)*

5.1.2 Country of birth: ☐ Australia ☐ Other, *(specify)*:

5.1.3 Gender: ☐ Male ☐ Female

5.2 Ethnicity Please tick ☑ one or more

- ☐ Oceanian: Australian or New Zealander
- ☐ Oceanian: Aboriginal or Torres Strait Islander
- ☐ Oceanian: Pacific Islander (except Maori)
- ☐ Oceanian: Maori
- ☐ North-West European
- ☐ Southern and Eastern European
- ☐ North African and Middle Eastern
- ☐ South-East Asian
- ☐ North-East Asian
- ☐ Southern and Central Asian
- ☐ Peoples of the Americas
- ☐ Sub-Saharan African
- ☐ Prefer not to answer

5.3 Religion Please tick ☑ one

- ☐ Buddhism
- ☐ Christianity
- ☐ Hinduism
- ☐ Islam
- ☐ Judaism
- ☐ No religion
- ☐ Other, *specify*______________________
- ☐ Prefer not to answer

5.4 Country completed pre-registration health professional training

Please specify:

5.5 Communication training Please tick ☑ all that apply

- ☐ Australasian Donor Awareness Program (ADAPT)
- ☐ Core workshop ***and** attendance* ☐ some or ☐ completed
- ☐ Practical workshop ***and** attendance* ☐ some or ☐ completed
- ☐ Simulation workshop ***and** attendance* ☐ some or ☐ completed
- ☐ Other, *specify*:
- ☐ Have not attended

5.6 Years worked in intensive care I__I |__I years **or** ☐ ≤ 1 year

5.7 Number of family donation conversations led in the last complete calendar year? I__I I__I

5.8 Designation Please tick ☑ all that apply

- ☐ RN: Donation Specialist Nurse
- ☐ MD: Donation Specialist Medical
- ☐ SW: Social worker
- ☐ Other, *specify:*
- ☐ MD: Intensivist
- ☐ MD: Registrar (ICU)
- ☐ MD: Resident (ICU)

5.9 Responsible for the potential donor's medical management while raising donation with the family?

☐ Yes ☐ No

Form 6: Potential donor details

6.1 Potential donor details

6.1.1 Date of birth: |__| |__| / |__| |__| / |__| |__| |__| |__| *(dd/mm/yyyy)*

6.1.2 Country of birth: ☐ Australia ☐ Other, *(specify)*:

6.1.3 Gender: ☐ Male ☐ Female

6.2 Was the potential donor's donation decision registered in life? Please tick ☑ all that apply

- ☐ Yes to donation *found on* ☐ AODR and/or ☐ RMS
- ☐ No to donation *found on* ☐ AODR and/or ☐ RMS
- ☐ Not registered/not found *registers checked* ☐ AODR and/or ☐ RMS
- ☐ Registers not accessed *because* ☐ infant/child or ☐ overseas resident

6.3 Ethnicity Please tick ☑ one or more

- ☐ Oceanian: Australian or New Zealander
- ☐ Oceanian: Aboriginal or Torres Strait Islander
- ☐ Oceanian: Pacific Islander (except Maori)
- ☐ Oceanian: Maori
- ☐ North-West European
- ☐ Southern and Eastern European
- ☐ North African and Middle Eastern
- ☐ South-East Asian
- ☐ North-East Asian
- ☐ Southern and Central Asian
- ☐ Peoples of the Americas
- ☐ Sub-Saharan African
- ☐ Missing from medical record

6.4 Religion Please tick ☑ one

- ☐ Buddhism
- ☐ Christianity
- ☐ Hinduism
- ☐ Islam
- ☐ Judaism
- ☐ No religion
- ☐ Other, *specify:*________________________
- ☐ Missing from medical record

6.5 Primary event/cause of death Please tick ☑ one

- ☐ Motor vehicle accident
- ☐ Motor bike accident
- ☐ Cyclist
- ☐ Pedestrian
- ☐ Other road accident
- ☐ Fall
- ☐ Other accident
- ☐ Gunshot
- ☐ Felony or crime e.g assault
- ☐ Other, *specify:*
- ☐ Spontaneous subarachnoid haemorrhage
- ☐ Other spontaneous intracranial haemorrhage
- ☐ Cerebral infarct
- ☐ Hypoxia
- ☐ Cerebral oedema
- ☐ Cerebral tumour, specify benign or malignant
- ☐ Drowning
- ☐ Hanging
- ☐ Asthma

6.6 Certification of death ☐ Brain death criteria ☐ Circulatory death criteria

Date |__| |__| / |__| |__| / |_2_| |_0_| |_1_| |__| *(dd/mm/yyyy)* Time |__| |__|:|__| |__|

6.7 Admission to this intensive care unit

Date |__| |__| / |__| |__| / |_2_| |_0_| |_1_| |__| *(dd/mm/yyyy)* Time |__| |__|:|__| |__|

***Forms 2-6 are complete:__________________________________ (DSN sign) ____________(date)**
